# The Effect of Depth on the Morphology, Bacterial Clearance, and Respiration of the Mediterranean Sponge *Chondrosia reniformis* (Nardo, 1847)

**DOI:** 10.3390/md18070358

**Published:** 2020-07-10

**Authors:** Mert Gökalp, Tjitske Kooistra, Miguel Soares Rocha, Tiago H. Silva, Ronald Osinga, AlberTinka J. Murk, Tim Wijgerde

**Affiliations:** 1 Marine Animal Ecology Group, Wageningen University and Research, P.O. Box 338, 6700 AH Wageningen, The Netherlands; tjitske.kooistra@wur.nl (T.K.); ronald.osinga@wur.nl (R.O.); tinka.murk@wur.nl (A.J.M.); tim.wijgerde@wur.nl (T.W.); 2 3B’s Research Group, I3Bs–Research Institute on Biomaterials, Biodegradables and Biomimetics, University of Minho, Headquarters of the European Institute of Excellence on Tissue Engineering and Regenerative Medicine, AvePark, Parque de Ciência e Tecnologia, Zona Industrial da Gandra, 4805-017 Barco, Guimarães, Portugal; miguelsoaresrocha@gmail.com (M.S.R.); tiago.silva@i3bs.uminho.pt (T.H.S.); 3 ICVS/3B’s–PT Government Associate Laboratory, 4806-909 Braga/Guimarães, Portugal

**Keywords:** sponge, osculum size, respiration, clearance rate, depth, *Chondrosia reniformis*, collagen, integrated multitrophic aquaculture

## Abstract

To support the successful application of sponges for water purification and collagen production, we evaluated the effect of depth on sponge morphology, growth, physiology, and functioning. Specimens of Eastern Mediterranean populations of the sponge *Chondrosia reniformis* (Nardo, 1847) (Demospongiae, Chondrosiida, Chondrosiidae) were reciprocally transplanted between 5 and 20 m depth within the Kaş-Kekova Marine Reserve Area. Control sponges at 5 m had fewer but larger oscula than their conspecifics at 20 m, and a significant inverse relationship between the osculum density and size was found in *C. reniformis* specimens growing along a natural depth gradient. Sponges transplanted from 20 to 5 m altered their morphology to match the 5 m control sponges, producing fewer but larger oscula, whereas explants transplanted from 5 to 20 m did not show a reciprocal morphological plasticity. Despite the changes in morphology, the clearance, respiration, and growth rates were comparable among all the experimental groups. This indicates that depth-induced morphological changes do not affect the overall performance of the sponges. Hence, the potential for the growth and bioremediation of *C. reniformis* in mariculture is not likely to change with varying culture depth. The collagen content, however, was higher in shallow water *C. reniformis* compared to deeper-growing sponges, which requires further study to optimize collagen production.

## 1. Introduction

Sponges are found at all latitudes, living in a wide array of ecosystems varying in temperature and depth [1,2]. They are filter-feeding organisms often dominating the benthos in terms of abundance and biomass [3,4,5,6]. Sponges process huge amounts of water daily at up to 900 times their body volume of water per hour and filter 50,000 liters of seawater per liter of sponge volume per day [7,8,9,10], which is comparable to well-established suspension feeders such as mussels [11,12,13]. Sponges have a high efficiency and capacity for particle retention [3,4,14], preferably small particles (<10 µm) such as bacteria [4,15,16,17,18], phytoplankton [3,19], viruses [20], and dissolved organic matter [21,22,23,24]. This efficient and versatile filtration makes sponges key drivers of the uptake, retention, and transfer of energy and nutrients within benthic ecosystems [25,26,27,28] and makes them interesting candidate species for the bioremediation of organic pollution, such as waste streams from aquaculture [29,30,31]. 

Around sea-based fish cultures, the elevated densities of bacteria and reduced oxygen levels caused by the excreta of cultured fish and uneaten fish feed negatively impact the seabed and add substantial pressure on the surrounding environment that could even affect the aquaculture and other ecosystem services [18,32,33,34,35]. However, the excess amount of bacteria in/around such an organically loaded culture system generates a potential food source for sponges [29]. In our recent study, we discovered that *Chondrosia reniformis* (Nardo, 1847) benefited from mariculture-sourced organic pollution and showed better a growth performance in polluted waters compared to a pristine environment (170% versus 79% in 13 months [31]). *C. reniformis* has been reported as a rich source of biomedically interesting types of collagen [36,37,38], which could be used in 3D printing inks for medical applications (i.e., scaffolds for bone and cartilage tissue regeneration [39,40]). This result suggests the potential for the utilization of this sponge in an integrated multi-trophic aquaculture (IMTA) system where it not only improves water quality but also produces collagen.

To optimize the combined use of sponges as producers of natural products and bioremediators, we need to assess the biomass production and associated natural product content and link these to the actual in situ filtration capacity of the model species. In the literature, different terms are used that relate to the filtration activity by sponges. In this paper, we will refer to the terms pumping, filtration, and clearance as follows: pumping is the volumetric water flow rate through the sponge body generated by the aquiferous system of the sponge; filtration and clearance are defined in the same way and refer to the amount of water per unit of time that is entirely cleared of a specific type of component, e.g., bacteria, microalgae, or dissolved organic matter (DOM). We use the term clearance usually in association with the measurements that are applied to determine the clearance/filtration rates. Pumping and clearance/filtration relate to each other through the retention efficiency for the specific component, which means that, for example, clearance rates for bacteria can be different from clearance rates for microalgae. Under in situ conditions in open water, the actual particle uptake rate for a sponge can be calculated by multiplying the ambient concentration by the clearance rate. In this case, a constant ambient particle concentration can be assumed because seawater around the sponge is replenished by water movement before the exhalant water can be inhaled for a second time.

To fully comprehend the role of sponges in the transfer of nutrients and energy in natural and artificial ecosystems such as IMTAs, it is important to understand the physiological and environmental factors that control their ability to take up specific components of interest out of the environment. Some of these factors have already been identified. For example, sponges hosting large quantities of associated microbes (high microbial abundance, or HMA sponges) including *C. reniformis* usually have lower pumping rates than species with low numbers of associated microbes [8]. Additionally, the elevated amounts of suspended sediment in the surrounding water reduce the sponge pumping activity [41]. The ambient flow and sponge morphology may influence the pumping and filtration rates of a sponge by either reducing the amount of energy that is needed for active pumping [42,43] or by altering the efficiency of retaining the particulate matter from the filtered water. Conflicting results have been reported on the relationship between temperature and filtration by sponges [14,44,45], and information on the effects of seasonality on pumping and filtration by sponges is scarce. Very recently, [46] conducted a comprehensive study spanning two annual cycles to assess the factors that regulate the in situ sponge pumping of five Mediterranean sponges (including *C. reniformis*). Unexpectedly, these authors reported no significant effect of temperature and particulate organic matter on the sponge pumping rates and no clear trend of seasonality. Instead, sponge size was found to be the main predictor of volumetric pumping rate, with larger sponges exhibiting a relatively lower pumping rate per unit of body volume. 

To date, no studies are available that investigate the role of water depth on sponge pumping and filtration. In maricultures, culture depth may vary between and within locations. The eventual effects of water depth on sponge pumping activity may influence choices for sponge culture sites and sponge culture methodology. In an earlier survey, we observed some physiological differences between *C. reniformis* populations from different depth zones [47]. The osculum density, osculum size, and the associated osculum outflow rate differed significantly between two depth groups (0–3 m and 20–25 m). The bacterial clearance, however, was not measured, so the functional consequences of the observed morphological differences for the filtration/bioremediation capacity of *C. reniformis* remain unknown [47]. Concurrent with potential effects on bacterial clearance and filtration efficiency, water depth may also affect other parameters that are relevant to sponge mariculture, such as sponge growth and the production of secondary metabolites and collagen. Therefore, the aim of this study was to investigate the effect of depth on the filtration capacity (measured as in situ bacterial clearance rates), metabolism (respiration rate as oxygen consumption), morphology (density and size of oscula), growth, and the collagen/biomass production of *C. reniformis*. Up to this date, there is no scientific work that combines the sponge growth performance and related biomaterial content with the natural pumping and filtration rates of sponges (except from 51, who focused on the effect of copper tolerance on the clearance rate). Thus, the current work represents a key step forward, both in terms of understanding the morphological plasticity and performance of sponges and in the application of *C. reniformis* in IMTA for combined bioremediation and collagen production.

## 2. Results

### 2.1. General Observations

The water temperatures and salinity values recorded at the site during the transplantation and incubation experiments ranged between 26 and 28 °C and 38.5 and 38.8 g L^−1^, respectively. The *C. reniformis* explants exhibited a swift recovery. Open surfaces had completely healed five days after sampling and cutting (Figure S1). The visible distinctive inner layers of the sponges were observed to change as early as from day one. A white layer was formed in and around the open surfaces, the cut marks completely disappeared, and the large gaps observed in the mesohyl of some explants were filled completely after 5 days. The explants rapidly reshaped their aquiferous structure, reorganizing the collagen-rich ectosome and mesohyl while regenerating the cut surfaces.

### 2.2. Natural Sponge Morphology

**Figure 1:**
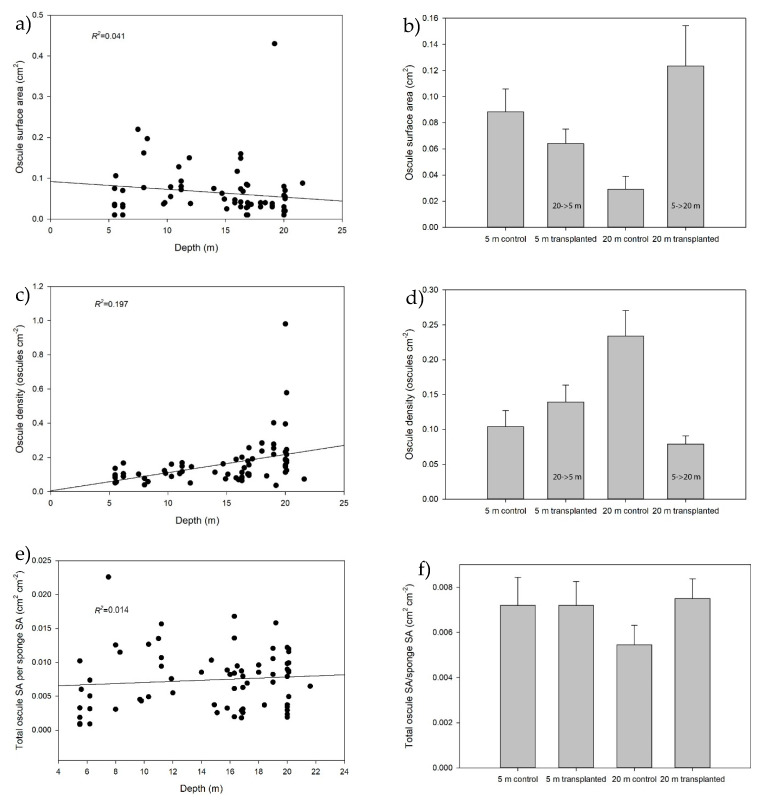
Natural sponges vs. Experimental Sponges. (**a**) Natural—significant decrease of average oscule surface area over depth (Pearson’s *r* = −0.245, *p =* 0.044). Dots represent individual sponges. (**b**) Transplanted—oscule surface area (cm^2^) measured 8 weeks after transplantation. Values are means + SEM, *N* = 8–10 per group (20 m control vs. 20 m transplanted *F* = 3.052, *p* = 0.005). (**c**) Natural—significant increase of oscule density over depth (Pearson’s *r* =0.444, *p* < 0.001). Dots represent individual sponges. (**d**) Transplanted—oscule density measured 8 weeks after transplantation. Values are means + SEM, *N* = 8–10 per group (5 m control vs. 20 m control, *F* = 3.052, *p* = 0.001; 20 m control vs. 20 m transplanted, *F* = 3.052, *p =* 0.000). (**e**) Natural—total oscule surface area (OSA) per unit of sponge surface area (SSA) of natural sponges. (Spearman’s rho = 0.117, *p* = 0.335). Dots represent individual sponges. (**f**) Transplanted—total oscule surface area (OSA) per unit of sponge surface (SSA) measured 8 weeks after transplantation. Values are means + SEM, *N* = 8–10 per group.

In sponges along a natural depth gradient, there was a significant decrease in the average osculum size with depth (Pearson’s *r* = −0.245, *p* = 0.044; Figure 1a) and a significant increase in the OD (osculum density) with depth (Pearson’s *r* = 0.444, *p* < 0.001; Figure 1c). No relationship between the depth and total OSA (osculum surface area) per sponge area was found for natural sponges (Spearman’s rho = 0.117, *p* = 0.335, Figure 1e). Hence, on average deep water sponges have more yet smaller oscula than shallow water sponges, but the total osculum surface per SSA (sponge surface area) remains the same with increasing depth.

### 2.3. Effect of Depth and Transplantation on Morphology

For OSA, significant differences between the four experimental groups were found (Table 1). Sponges transplanted from 5 m to 20 m did not reduce their osculum size and had significantly larger oscula compared to the 20 m control explants (Table 1). In contrast, the sponges transplanted from 20 m to 5 m did increase their osculum size, resulting in no significant difference with the 5 m control group (Figure 1b and Table 1). For OD, a similar pattern was found, as sponges transplanted from 5 m to 20 m did not alter their morphology and thus had significantly different ODs from the 20 m control sponges (Figure 1d and Table 1). Sponges transplanted from 20 m to 5 m matched the OD of the 5 m control sponges, with no significant difference found between these groups (Figure 1d and Table 1). One observation to note is the effect of depth on the non-transplanted control sponges. Although not significant here (Table 1), there seems to be a considerable osculum size difference between the 5 and 20 m control groups (0.9 vs. 0.3 cm^2^, respectively, Figure 1b). In addition, there is a highly significant difference (Table 1) in the OD between the groups (0.10 vs. 0.24 oscula cm^−2^, respectively, Figure 1d). Thus, the osculum size is about three times larger at 5 m, whereas the OD is approximately two times lower, which matches the observations on the natural sponges (Figure 1a,c). For the total OSA per sponge area, no significant differences were found between the experimental groups, again in line with natural sponges (Figure 1e,f and Table 1). 

### 2.4. Clearance and Respiration Rates

The mean clearance rates of the experimental sponges were 136.3 ± 21.09 mL cm^−3^ h^−1^ (Table 2). No significant effects of depth and transplantation on the clearance rates were found (Table 1). The respiration rates, only measured at 5 m due to technical limitations, were 0.07 ± 0.01 mg O_2_ cm^−3^ h^−1^ (Table 2) and did not differ between the transplanted and control sponges (Table 1).

### 2.5. Survival and Growth Rates 

Both the control and transplanted sponges readily adapted to their new habitat on the incubation plates. Two sponges from the 5 m control group were lost; they disappeared due to unknown causes, but the rest of the explants (*N* = 38) remained at the center of the plates, where they were initially attached and a 95% survival was achieved (Table 2). The overall growth rate was 63.6 ± 5.4% after 8 weeks in culture (Table 2) without significant effects of depth and transplantation (Table 1).

### 2.6. Collagen Quantification

The collagen yields of the control and transplantation groups are shown in Table 3. The highest extraction yield was observed for the 5 m control group (35.5% of wet mass), whereas the 20→5 m transplanted group had the lowest collagen content (14.5%). The 20 m control and 5→20 m transplanted groups revealed similar collagen yields (18.4% and 21.6%, respectively). 

## 3. Discussion

The aim of this study was to investigate the effect of depth on the bacterial clearance, morphology, respiration, growth, and collagen production of *C. reniformis*. Our data show that the osculum morphology is depth-dependent, whereas the bacterial clearance rate, respiration, and growth are not. The collagen content also seems to be affected by depth, which will be further elaborated on below.

### 3.1. Morphology and Bacterial Clearance Rates in Relation to Depth 

Along a natural depth transect, both OSA and OD significantly correlated with depth in a reciprocal way, with deeper living sponges having more but smaller oscula. These trends are in line with our earlier survey [47] and with measurements on non-transplanted experimental sponges in the current study. In the earlier survey, the average OSA of sponges in shallow water (0–3 m) was 0.71 cm^2^ compared to 0.05 cm^2^ in deeper water (20–25 m)—i.e., 14-fold larger. In the current study, the average OSA of the non-transplanted sponges was 0.09 cm^2^ at a 5 m depth versus 0.03 cm^2^ at a 20 m depth, and so was three-fold larger. The most prominent difference with our previous study was the almost eight times smaller OSA, which may relate to the difference in depth of the “shallow” sponges (0–3 m versus 5 m). We hypothesize that in more shallow water, effects of wave action and the concurrent resuspension of larger particles (e.g., course sand, small pebbles) are much greater than in deeper waters. In order to prevent clogging of their oscula by these particles, sponges in shallow waters need stronger pumping forces to remove these particles from their oscula, which is favoured by fewer but larger oscula. This effect may become most explicit in the surf zone (0–3 m), where wave forces are maximal.

In the current study, the total OSA:SSA ratio remained constant over depth, both in sponges growing along a natural depth gradient and in the non-transplanted experimental sponges at two depths. Hence, the sponges apparently compensate for the increase in OSA by making less oscula, thus maintaining a similar total pumping capacity. We conclude that *C. reniformis* shows morphological plasticity over depth that leads to a depth-independent capacity to filter bacteria from the surrounding water.

Interestingly, the morphological response of *C. reniformis* to transplantation differed between the two transplantation treatments. In agreement with sponges growing along a natural depth gradient, we found that transplantation of sponges from 20 to 5 m resulted in morphological changes, with an increase in OSA and a decrease in OD. Thus, deeper-growing sponges transplanted from 20 to 5 m adapted their morphology to match that of shallow-water individuals. However, the sponges transplanted from 5 to 20 m retained their original morphology. This suggests that the conditions in the wave action area immediately trigger the sponges to adapt their morphology, as described above. Additionally, the higher collagen content of the shallow sponges (Table 2), which makes them more robust, suggests an adaptation to the greater forces imposed on them. Another explanation for the lack of morphological adaption in the deeper sponges is that 8 weeks was insufficient time for the shallow-water sponges to reorganize their aquiferous system. Our own observations on fast healing after the cutting of explants, (Figure S1) and previous findings on morphological plasticity do not support this explanation. In *Dysidea avara*, the closure of oscula and formation of new ones occurred within days to weeks [43]. Moreover, many sponges, including *C. reniformis*, show very fast cell cycling [22] and rapid wound healing [48,49,50]. An alternative explanation for the lack of response of sponges transplanted to 20 m relates to the hypothesis outlined above, which suggests that the observed morphological plasticity relates to the wave action and corresponding resuspension of larger particles. Since sponges at 5 and 20 m depths showed a similar capacity to clear bacteria, there may be no immediate trigger for the replumbing of the aquiferous system in sponges transplanted from 5 to 20 m, whereas sponges transplanted from 20 to 5 m would have to modify their aquiferous system to cope with the higher coarse sediment loadings. It remains to be investigated whether the sponges transplanted to deeper waters will increase their OD and decrease their OSA over a longer period. To investigate whether and how the internal aquiferous system is affected by depth, histology is recommended for future experiments.

This study is the first to report on a combination of in situ bacterial clearance rates and respiration rates for *C. reniformis*. No earlier data on in situ respiration in this species are available in the literature. The mean respiration rate of 0.07 mg O_2_ cm^−3^ h^−1^ (equal to 2.2 ± 0.1 µmol O_2_ cm^−3^ h^−1^) measured in our study falls in the middle of the range of respiration rates for various other sponge species [7,51]. The in situ clearance rates for *C. reniformis* have previously been reported [52] to range from 50 to 340 mL h^−1^, which appears to be in good agreement with the mean value of 136.3 mL cm^−3^ h^−1^ reported here, but unfortunately [52] did not indicate whether the clearance rates had been normalized to a biomass parameter. Possibilities to compare our clearance data with the literature values for other species are limited due to the variation in the units in which clearance rates are expressed. We normalized the rates obtained in this study to both sponge volume (cm^3^) and mg DM, using a conversion factor of 5.68 to convert the volume to DM (Table S1) to enable multiple comparisons. A comparison with the literature data (Table 4) shows that, similar to the respiration rates, the clearance rates measured for *C. reniformis* are also in the middle of the range reported for Mediterranean sponge species. *C. reniformis* is categorized as a High Microbial Abundant or HMA sponge [53]; these are species that host high numbers of bacteria in their bodies. HMA sponges tend to have lower pumping rates than Low Microbial Abundant (LMA) sponges [8]. The bacterial clearance rate reported here (136.3 mL cm^−3^ h^−1^) is in the same order of magnitude as the average pumping rate of ~300 mL cm^−3^ h^−1^ reported for HMA sponge species (reviewed in [8]). Since clearance rates are always lower than volumetric pumping rates (the difference depending on the retention efficiency of the targeted particles), we conclude that our data are well in line with the literature values.

The *C. reniformis* clearance rates were considerably higher than those found for Mediterranean *C. nucula* [15], a closely related species. This may relate to the type of particles that were used for the incubation experiments. To detect clearance in *C. nucula*, cultures of *Escherichia coli* were used [15], which may not resemble the natural populations of bacteria in seawater. All the other studies referred to in Table 4 used natural food assemblages and show considerably higher but also highly variable rates. Sponges can exhibit differential clearance rates for different categories of picoplankton occurring within natural assemblages, and clearance rates can change over the season [55]. Comparative studies such as the current study on the effect of depth should therefore be performed using standardized methodology (i.e., always using the same target particles) within a short time frame.

### 3.2. Survival, Growth, and Collagen Content

Culturing *C. reniformis* explants has always been problematic as a result of the extreme motility of this species, making them “escape” from the intended location [29,30,31,57,58,59]. However, the custom design incubation plates with a protective PVC rim (poly vinyl chloride) preventing them from escaping and a protective chicken wire lid to prevent predation as applied in this study proved to be successful. The setup provided easy handling for clearance rate experiments and growth measurements. The main challenge was the pinning of explants onto the nails during outplanting due to the contraction of the sponges following initial disturbance (sampling and cutting), making their tissue very hard. When this succeeded, attachment was successful and the explants remained in place. The survival rate we achieved (95% after 8 weeks of culture) is considerably higher than the survival in our previous study, 39–86% after 56 weeks [31], or 55% in a study by [59]. In the study reported by [30], the protective stainless-steel cage around explants of *C. reniformis* resulted in comparable survival rates for explants cultured under pristine conditions. 

In line with the similar pumping capacity, clearance rates, and respiration rates across the treatment groups, we also did not observe growth differences. This has implications from the perspective of integrated multitrophic aquaculture (IMTA) and bioremediation, as it does not seem to matter at what depth the *C. reniformis* explants are placed in terms of performance. The growth rates observed in this study (52–83% in two months) are in line with our previous results—70–114% in 6 months [31]. In spite of the variable growth rates and peculiarities (growing protrusions, dripping through meshes, etc.) reported by earlier studies [29,57,59], our current and previous studies show that *C. reniformis* remains a good candidate for mariculture [30,31]. Although the growth rate of this species is not as high as that reported for other species such as *Mycale hentcheli* (359–2437% year 1 [60]) or *Lissodendoryx* sp. (5000% month 1 [61]), the high survival rates and reproducible growth rates obtained when applying appropriate methods will enable the controlled production of *C. reniformis* biomass through sea-based aquaculture.

Clearly, *C. reniformis* is a potential source of collagen for biomedical applications in tissue engineering and regenerative medicine because of the unique physicochemical properties of the collagen and the high collagen content [62,63,64,65]. Our results are in line with earlier studies that quantified collagen in *C. reniformis*. Swatschek et al. [63] determined a collagen content of 30% of the freeze-dried mass of *C. reniformis*. Considering a wet mass to dry mass ratio of 5.68 (Table S1), this would translate into a collagen yield of 5.3% of the wet mass. More recently, Pozzolini et al. [37] found a similar value (355 mg collagen in 998 mg of dry weight, which equals 30%) using the methodology that was also applied in the current paper. Our yield of 14.5–35.5% wet mass (Table 3) is much higher. Possibly, the fact that we removed excess water during the homogenization of the sponge before weighing may have resulted in a relatively lower wet mass and thus a relatively higher wet mass-based collagen content. The results show, however, that the cultured materials and the methodology to extract collagen are suitable to collagen for pilot-scale processes. Additionally, the developed approach enables further collagen yield optimization, e.g., via the further investigation of genotype-specific yields and the effects of specific depths and other environmental variables on collagen content. 

In conclusion, the filtration capacity, metabolism, and biomass production of *C. reniformis* are not affected by depth, in contrast to morphology and collagen content. Osculum morphology clearly is depth-dependent, where sponges transplanted from 5 m to 20 m reflect the conspecifics at their origin (do not adapt), whereas sponges transplanted from 20 m to 5 m reflect the conspecifics at their destination (adaptation). This adaptation to shallow water might relate to wave action and sediment loading. The sponges maintain their filtering capacity by presumably re-shaping their aquiferous system depending on their needs. This maintained filtering capacity is very promising for the future multifunctional application of *C. reniformis* to improve water quality by filtering out several types of organic pollution, including feces and unused feed from fish farms and pollution and microorganisms from urban sewage outlets. 

## 4. Materials and Methods 

### 4.1. Study Location and Seawater Parameters

**Figure 2:**
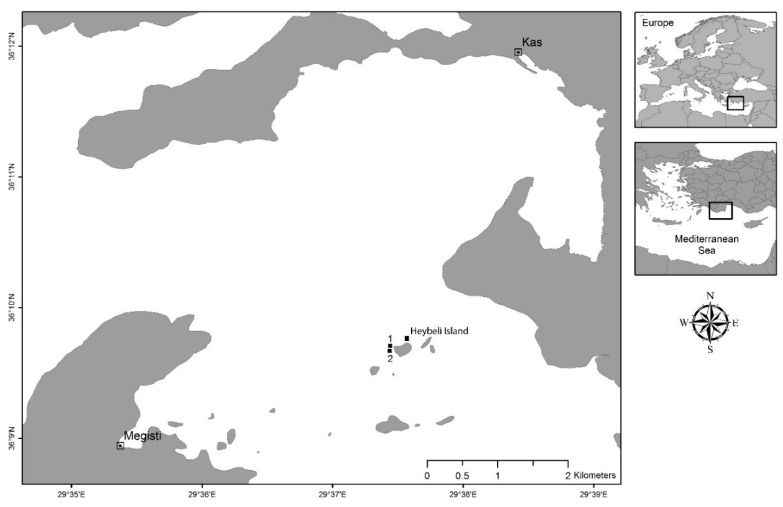
Map of Kaş-Kekova Special Environmental Protected Area. Pina Reef diving location is located at the eastern tip of Heybeli Island. The exact locations of experiments; (1) Pina reef wall (20 m depth), (2) Pina small reef (5 m depth) (36°09′42.4″ N 29°37′26.5″ E; 36°09′40.2″ N 29°37′26.3″ E; respectively).

The study was conducted over a period of 79 days during July–October 2018 at Pina Reef, a location within the Kaş-Kekova Special Environmental Protected Area (SEPA), Turkey (Figure 2). Pina Reef is located at the eastern side of Five-Islands, 4.3 km south of Kaş, and is exposed to wind and wave action originating from the open sea (west direction). The Pina reef wall is located right next to a 350 m-long wall structure located transversely Northwest–Southeast between 14 to 32 m depth. The Pina small reef, located 70 m south of the Pina reef wall, is a reef shoal with a depth ranging from 3.8 to 22 m, with adjacent Posidonia sea grass meadows in the south and east directions. The water temperature and salinity during the study period were measured by a multimeter during the clearance rate and respiration analysis (Multi 3620 IDS with TetraCon 959 and FDO 925 sensors, WTW, Weilheim, Germany).

### 4.2. Sponge Collection, Seeding, and Transplantation 

**Figure 3:**
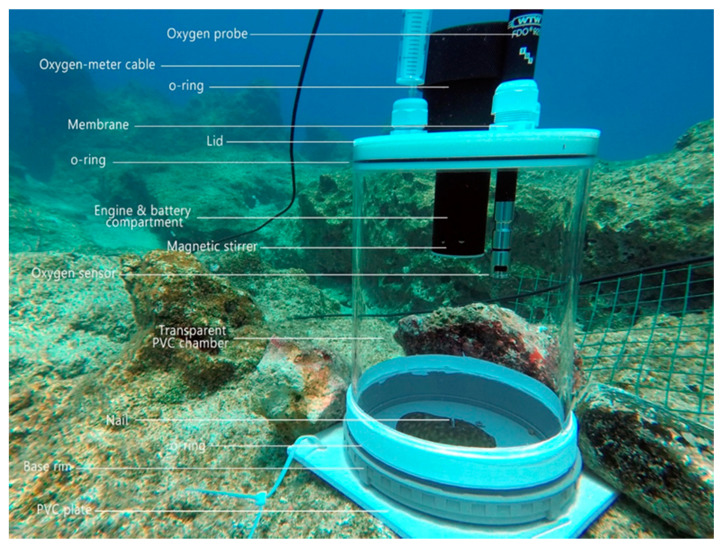
Incubation chambers used in bacterial clearance and respiration experiments. The oxygen probe was connected to a surface multimeter with a Kevlar-protected 20 m cable (WTW, Germany). Four O-rings ensured a water-tight incubation chamber. The first one was located around the black engine and battery compartment, sealing the lid. The second one was in the oxygen sensor lid. A third one was located around the white lid, sealing the transparent acrylic chamber. The final one was located inside the grey base-rim, sealing the chamber. At the end of the black engine and battery compartment, a magnetic stirrer was located.

The sponge specimens for the experiments were collected from 5 and 20 m (20 per depth, 40 in total), cut into pieces of 3–4 cm following the method described in Gökalp et al. [31] and fixed onto grey PVC plates with a rim with a radius of 17 cm and 2.7 cm high. Each PVC plate had a 15 cm-high cylindrical protective PVC rim (Figure 3) and was covered with 2 cm mesh size chicken wire in order to eliminate predation from larger marine life (e.g., sea turtle intrusion on the sponges in culture with boxes lacking protection; personal observation). The protective rim also prevented the sponges from migrating off the plate and prevented them from being carried away by occasional strong water currents [31].

**Figure 4:**
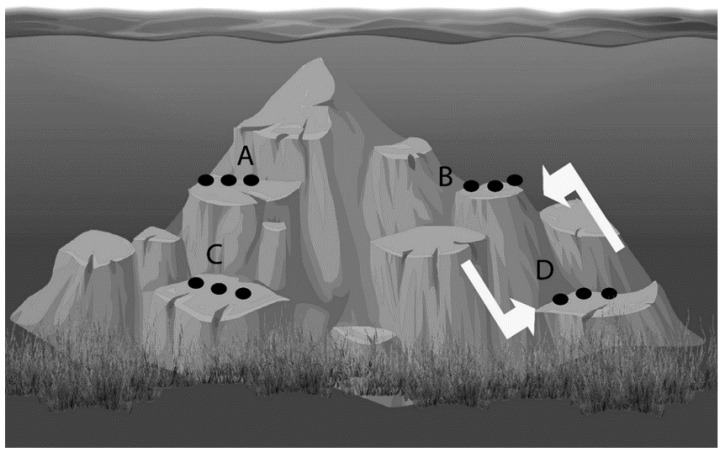
Overall view of the transplant and control groups: Group A—5 m control; Group B—5 m transplanted; Group C—20 m control; Group D—20 m transplanted. For the experiment, 10 specimens from 5 m moved to 20 m, and 10 specimens from 20 m moved to 5 m (50 m distance to each other). The other remaining sponges were kept at their depths as control, resulting in four groups of *N* = 10 sponges per group.

Each plate carried a chromium nail at the center with the sharp edge facing upwards (4 cm in height) to secure the specimens to the plate. After fixing the explants on pre-labelled PVC plates, they were left at their respective depth of origin (either 5 or 20 m) for 5 days to provide sufficient time to recover and attach to the PVC plates [49]. Subsequently, the PVC plates were transplanted by moving 10 plates from 5 to 20 m depth and 10 plates from 20 to 5 m depth (Figure 4; *N* = 10). Following transplantation, the sponges were left to acclimatize for seven days before the incubations started.

### 4.3. Determination of Sponge Size, Growth, and Morphological Characteristics

The size of the sponges and the number and size of the oscula were measured following the acclimatization period. Simultaneously, the sponges were photographed from the top and four sides with a ruler for scale using a Canon Eos 5D Mark IV camera and an Ikelite 5D housing setup. The sponge surface area (SSA) was measured from the projected surface area based on a picture taken from the top using the ImageJ software. The pictures from the sides were used to determine the average height of the sponge. Multiplying the measured average height with the SSA provided the approximate volume of the sponge (Vsponge), assuming a cylindrical shape. At the end of the experimental period, 5 sponges per group were weighed (wet mass, WM) and the volume was measured by water displacement to determine the accuracy of the volume calculation. To determine the wet mass (WM) to dry mass (DM) ratio, 6 individuals of the same species that were not used for the experiment were weighed, dried, and reweighed (Table S1). These size measurements were used (1) to determine the growth (using $\frac{t1-t0}{t0}\times 100\%$ to calculate the % increase in SSA over 8 weeks [31]) and (2) to normalize the metabolic rates to a biomass parameter (volume). The average osculum size (hereinafter referred to as the osculum surface area (OSA), sponge surface area (SSA), and osculum density (OD, the number of oscula per unit of sponge surface)) were determined for each of the 40 experimental sponges, hereby using the pictures taken for size measurements. From these parameters, the osculum number to sponge size ratio and the ratio of the oscular surface area to sponge surface area (OSA/SSA) were calculated. In addition, the multiplication of the OD with OSA provides the pumping potential of a sponge by expressing the total OSA per SSA. For comparison, the same parameters were determined for a series of C. *reniformis* specimens occurring along a natural depth gradient ranging from 2 to 25 m in depth with scuba diving at several sites within 1 km of the experimental locations.

### 4.4. Clearance and Respiration Rates

The circular rims on the square PVC plates (Figure 3) were designed to fit airtight to a transparent PVC chamber with a 6.75 L inner volume and were specifically developed for in situ clearance and respiration measurements (Wageningen University, Gelderland, The Netherlands). The upper side of the cylinder could be closed with a lid in which a magnetic stirrer with a battery pack (Jansen Tholen B.V., Tholen, The Netherlands) was mounted. The stirrer could create sufficient water circulation and the continuous mixing of seawater in order to equalize the oxygen distribution within the cylinder and prevent the particles from settling onto the bottom plate. Each lid also was fitted with two diaphragms for water sampling and an opening for an oxygen probe (WTW, Germany). Together, the PVC plate with rim, cylinder, and lid formed a water-tight incubation chamber (Figure 3). During the incubation experiments, the protective cylinder and lid of chicken wire were removed and replaced by the water-tight incubation chamber setup. The chambers were applied for the simultaneous in situ measurement of the bacterial clearance and respiration of the experimental sponges. Eight weeks after transplantation, all 40 specimens in the experiment were incubated to determine their bacterial clearance and respiration rate. A total 10 empty PVC plates were incubated to determine the background activity not associated with the sponges. Prior to the incubations, the chicken wire and the protective rim were carefully removed from the bottom plates, thus minimizing stress to the sponge specimens. The deposited sediment and live organisms accumulated on the O-rings and PVC plates were removed with a toothbrush and no organic matter was left on the PVC plate inside the base rim. Then, the cylinders were secured onto the PVC plates and left to acclimatize for 15 min before the incubations. After the acclimatization period, the chamber lid was closed, and using a syringe the first 10 mL water sample was taken directly (t0) and a second 10 mL water sample was taken 15 min later (t1). A total of 15 min was deemed sufficient time to obtain reliable results for both the oxygen depletion and bacterioplankton decrease, considering the size of the containers and the reported bacterial grazing rates and respiration rates from the literature [15,49,64]. The collected water samples were labelled right after surfacing from the dive, immediately fixed with 0.57 mL of 35% formaldehyde solution to a final concentration of 2–4%, and placed in ice containers. At the end of each incubation day, the fixed samples were filtered over white 25 mm diameter 0.2 μm polycarbonate membranes (GE Osmonics, Minneapolis, MN, USA), which were structurally supported by a 0.45 μm GF–F-type membrane (Whatman International Ltd., Maidstone, England, UK). Subsequently, the filters were air-dried in the laboratory for at least 15 min and stored at –20 °C in Eppendorf tubes until further use. To determine the total bacterial counts, the filters were placed on a microscopic slide and 10 µL of DAPI mix was added to stain the bacteria present, as described previously in de Goeij et al. (2007). The bacterial numbers were determined based on pictures taken under a fluorescence microscope (Leica DM6 B; Leica Microsystems, Wetzlar, Germany). Per filter, 10 images were taken using the Leica-LasX software (V3.3.0. Leica Microsystems. Wetzlar, Germany) and a DFC365 FC camera (Leica Microsystems, Germany). From the pictures, the average bacterial numbers were determined by counting 10 fields or up to a maximum number of 250 bacteria by using ImageJ. The clearance rates were calculated using the following formula (modified from [56]):

(1) $$CR=V_{chamber}\cdot V_{sponge}{}^{-1}\cdot \left(rate\;sponge-rate\;blank\right),$$

in which *V_chamber_* stands for the volume of water in the incubation chamber (milliliter) and *V_sponge_* is the volume of the sponge. The rate of bacterial concentration change for the sponge (rate sponge) and the blank chambers (rate blank) is calculated as:

(2) $$\mathrm{Cell}\;\mathrm{change}\;\mathrm{rate}=\frac{\ln\left(C0\right)-\ln\left(Ct\right)}{t},$$

in which *C0* and *Ct* are the concentration of counted bacteria per milliliter at (t0) and (t1) and *t* is the total incubation time (15 min). 

To determine the respiration rates, the oxygen concentrations in the chambers were measured during the incubations with a Multi 3620 IDS (WTW, Germany) connected with 20 m cables to two FDO925 probes (WTW, Germany). During the incubations, the oxygen values were logged every 10 s from a boat, which was anchored right on top of the divers to release a sufficient amount of cable underwater. The respiration rates were only measured for the sponges at 5 m depth due to the limited reach of the WTW cables and prolonged diving times. Linear graphs were fitted to the measurements, and the respiration rates were calculated with the slope of the graphs, which represent the oxygen decline in mg O_2_·L^−1^·min^−1^. The respiration rates (RR) were calculated with the following formula:

(3) $$RR=\left(O_{2}\;slope\;sponge-O_{2}\;slope\;blank\right)\cdot V_{chamber}\cdot V_{sponge}{}^{-1}.$$

### 4.5. Collagen Extraction

After 8 weeks, 3 to 5 sponges from each of the four experimental groups were randomly collected and immediately frozen at −18 °C following the dives. The frozen samples were transported in dry ice containers to the facilities of University of Minho, Portugal, and kept at −20 °C until further analysis. All the procedures described below were performed separately with sponges pooled per experimental treatment to obtain enough material for the analysis. The samples were thawed and any exogenous materials on the sponges were removed by rinsing in dH_2_O. All the steps for collagen extraction were carried out at 4 °C. Next, the wet weight was determined and the sponges were cut into small pieces of roughly 1 × 1 × 1 mm. Excess water was poured from the marine sponge samples, and 5 sponge volumes of disaggregating solution (50 mM Tris–HCl buffer pH 7.4, 1M NaCl, 50 mM EDTA and 100 mM 2-mercaptoethanol) were added and left under stirring for 4 days. The collagen solutions (CS) were filtered with a nylon mesh to remove any remaining undissolved fragments and the solution was extensively dialyzed in dialysis tubing cellulose membrane for 7 days with 2 dialyzing buffer changes every per day (CS/dialyzing buffer ratio 1:1000) against dH_2_O to remove all traces of 2-mercaptoethanol. The suspensions were centrifuged for 10 min at 1200 g to further remove cell debris and sand particles. To collect the collagen from the suspension, another centrifugation followed for 30 min at 12,100 g, yielding pellets containing collagen which then were resuspended in dH_2_O. The collagen re-extraction was performed by repeating the second centrifugation step. The collagen solutions were stored at 4 °C. The total collagen content was determined by freeze-drying and weighing (dry mass) the extracted material.

### 4.6. Collagen Quantification

Following the collagen extraction, the obtained collagen solutions were analyzed regarding the collagen content. To determine the total amount of collagen extracted, each collagen solution was freeze-dried and then weighted (dry mass). The wet collagen extraction yield was determined using Equation (4):

(4) $$Yield\;of\;collagen\;\left(wet\right)\;\left(\%\right)=\frac{Mass\;of\;collagen\;\left(mg\right)}{Wet\;mass\;C.\;reniformis\;\left(mg\right)}\;)\times 100.$$

### 4.7. Data Analysis

All the data were tested for the normality and homogeneity of variances. A one-way ANOVA was performed to test for differences between the four treatment groups in terms of sponge morphology, clearance rate, and growth. The respiration rates of the transplanted and control sponges at 5 m depth were also compared with a one-way ANOVA. Planned contrasts with Bonferroni correction (i.e., using a corrected α for significance) were used to follow-up significant ANOVAs. Pearson’s product-moment correlations were used to correlate the osculum size and density of the natural sponges with depth, whereas Spearman’s rho for the total oscula surface area (SA) per unit of sponge surface area (SA) was used when the data were not normally distributed. A statistical analysis was performed using SPSS 25 (IBM SPSS Statistics for Windows, Version 25.0. Armonk, NY, USA: IBM Corp.); graphs were plotted with Sigmaplot 12 (Systat Software, San Jose, CA, USA).

## Figures and Tables

**Table 1:** One-way ANOVA testing all four experimental groups for differences in the oscule surface area, oscule density, % sponge growth in 68 days of culture, and clearance rate, along with one-way ANOVA testing for the effect of transplantation on the respiration rates (*N* = 8–10).

Variable	F/t	df	Error	*p*
*Oscule surface area (OSA)*				
	3.391	3	30	0.031 *
*Planned contrasts with Bonferroni correction*				
5 m control versus 20 m control	1.817	1	30	0.079
5 m control versus 5 m transplanted	0.811	1	30	0.424
20 m control versus 20 m transplanted	3.052	1	30	0.005 **
*Oscule density (OD)*				
	7.322	3	32	0.001 **
*Planned contrasts with Bonferroni correction*				
5 m control versus 20 m control	−3.557	1	32	0.001 **
5 m control versus 5 m transplanted	−1.012	1	32	0.319
20 m control versus 20 m transplanted	−4.464	1	32	0.000 ***
Total oscule surface area per unit of sponge surface area				
	0.821	3	31	0.492
*Clearance rate*				
	0.199	3	30	0.896
Respiration rate ****				
	0.143	1	15	0.711
*% growth*				
	1.447	3	32	0.248

* *p* < 0.050, ** *p* < 0.010, *** *p* < 0.001, **** respiration rate measured at 5 m only.

**Table 2:** Survival, growth, clearance, and respiration rates (%) of experimental sponges measured after 8 weeks in culture. Values are means + SEM.

Transplantation Experiment	Pooled	5 m Control *N* = 8	5 m Transplant. *N* = 10	20 m Control *N* = 10	20 m Transplant. *N* = 10
(8 weeks in culture)					
Survival Rates (%)	95	80	100	100	100
Growth Rates (%)	63.6 ± 5.4	83.0 ± 13.2	52.5 ± 8.3	60.3 ± 11.1	70.2 ± 10.4
Clearance Rate (mL cm^−3^ h^−1^)	136.3 ± 21.1	164.8 ± 43.9	120.8 ± 53.3	131.4 ± 32.9	136.5 ± 43.9
Respiration Rate (mg O_2_ cm^−3^ h^−1^) *	0.07 ± 0.00	0.06 ± 0.00	0.07 ± 0.01		

* Due to the cable reach only measured at 5 m depth.

**Table 3:** Total collagen content (% WM) of control and transplanted sponges after 64 weeks (*N* = 1 per group).

Treatment	Collagen Yield (%)
5 m control	35.5
5 m transplanted	14.5
20 m control	18.4
20 m transplanted	21.6

**Table 4:** Clearance rates for Mediterranean sponges in literature. DM = Dry Mass.

Species	Method (Incubation)	Sponge Size #	Clearance Rate	Reference
Ex situ				
*Crambe crambe*	0.25 h	5 cm^2^	506–790 mL g^−1^ DM h^−1^	Turon et al. 1997, [54]
*Dysida avara*	0.25 h	5 cm^2^	1380–3804 mL g^−1^ DM h^−1^	Turon et al. 1997, [54]
*Chondrilla nucula*		25 cm^3^	0.2 & 1.4 mL cm^−3^ h^−1^	Milanese et al. 2003, [15]
*Spongia officinalis*	4 h, 1 L	91.4 cm^3^	34–210 mL g^−1^ DM h^−1^	Stabili et al. 2006, [17]
*Corticium candelabrum*	1 h, 1 L,	0.13–18.8 cm^2^	1000–10.000 mL g^−1^ DM h^−1^	de Caralt et al. 2008, [55]
In situ				
*Dysida avara*	1 or 3 L, 4 h	25 cm^3^	104–2046 mL g^−1^ DM h^−1^	Ribes et al. 1999a, [14]
*Chondrosia reniformis*	4 L, 1 h		50–340	Cebrian et al. 2006, [49] *
*Ircinia variabilis*	1.5 h, 1 L	50 cm^3^	15.96 mL cm^−3^ h^−1^	Ledda et al. 2014, [56]
*Agelas oroides*	1.5 h, 1 L	35 cm^3^	20.0 mL cm^−3^ h^−1^	Ledda et al. 2014, [56]
*Chondrosia reniformis*	0.25 h, 6 L	9–49 cm^2^	136.3 mL cm^−3^ h^−1^ 639 mL g^−1^ DM h^−1^	This study

* Unit not specified in the study; # sponge size is expressed either as volume (m^3^) or as projected surface area (m^2^).

## Supplementary Materials

The following are available online at https://www.mdpi.com/1660-3397/18/7/358/s1: Figure S1: Healing phases of *C. reniformis* specimens following the sampling/cut; Table S1: Volumes, WM and DM from 6 randomLy collected C. *reniformis* specimen.
