# Supplementary material for: The Effect of Depth on the Morphology, Bacterial Clearance, and Respiration of the Mediterranean Sponge Chondrosia reniformis (Nardo, 1847)
Source: Mar Drugs. 2020 Jul 10;18(7):358. doi: 10.3390/md18070358 (PMC7401281; doi:10.3390/md18070358)
Supplement: Supplementary file 1 [file marinedrugs-18-00358-s001.pdf]

Supplementary
The Effect of Depth on the Morphology, Bacterial Clearance, and Respiration of the Mediterranean Sponge *Chondrosia reniformis* (Nardo, 1847)

Mert Gökalp [1,*], Tjitske Kooistra [1], Miguel Soares Rocha [2,3], Tiago H. Silva [2,3], Ronald Osinga [1], AlberTinka J. Murk [1] and Tim Wijgerde [1]

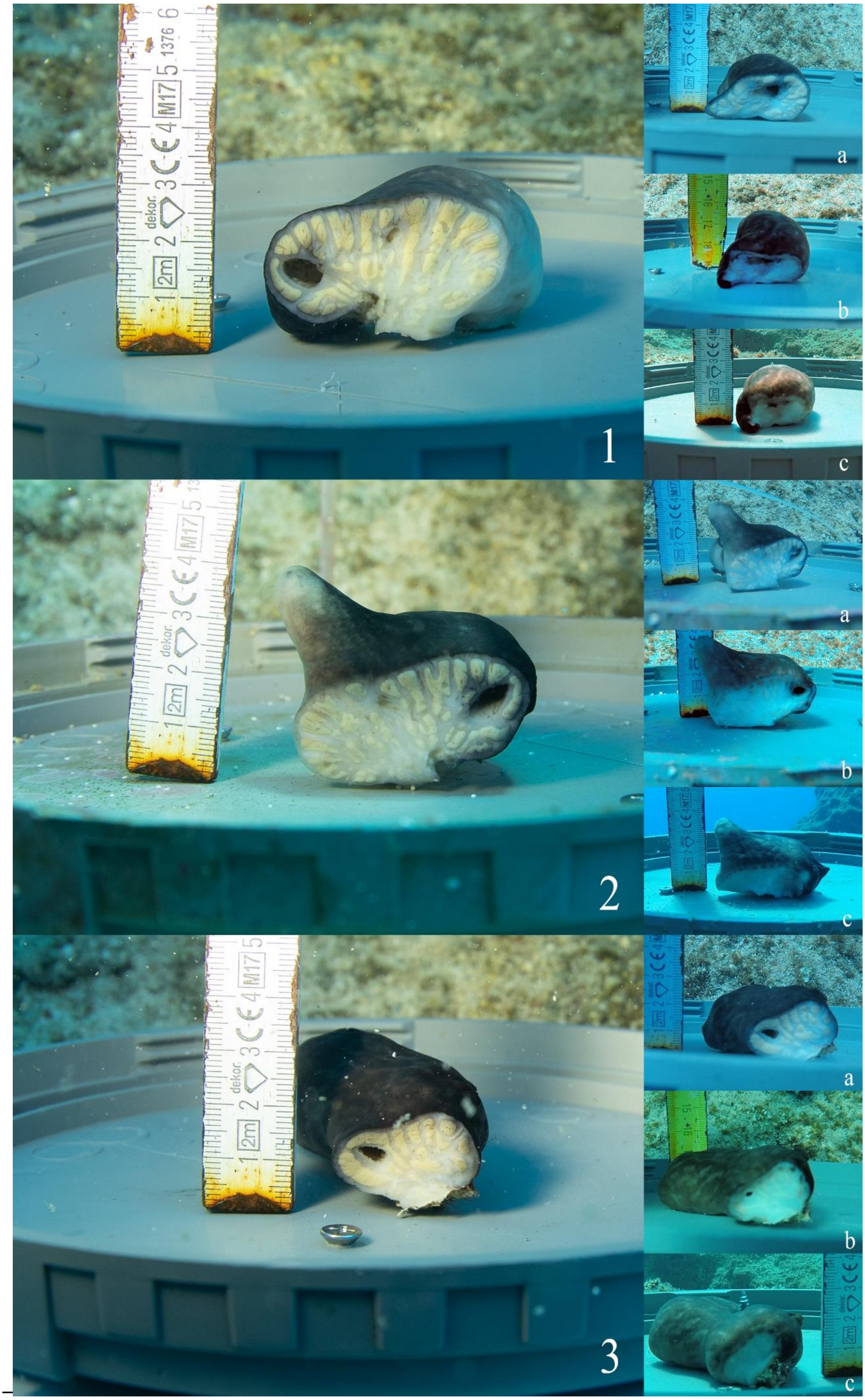

Figure S1. Healing phases of *C. reniformis* specimens following the sampling/cut. The larger background pictures were taken at Day 1 (specimen 1, 2, 3), right after the incision. The smaller pictures were taken at Day 2, Day 3 & Day 5 (top to bottom; a, b, c) respectively. The explants open surfaces observed to be

completely healed five days after being cut. The damages caused from incision are covered promptly, the holes are minimized and ectosome layer of the sponge observed to cover the damaged areas.

Table S1 Volumes, WW and DW from 6 randomly collected *C. reniformis* specimen.

Sponge Specimen	V calculated (cm^3)	V measured (cm^3)	WW (g)	DW (g)	WW:DW	Vm:DW	Vc:DW
1	17	20	21	4	5.25	5.0	4.32
2	24	25	28	4	7.0	6.25	6.10
3	32	25	33	5	6.6	5.0	6.31
4	40	30	42	8	5.25	3.75	5.03
5	39	38	43	8	5.38	4.75	4.84
6	21	17	23	5	4.6	3.4	4.25
mean					**5.68**	4.69	5.14
St.dev.					0.92	1.02	0.88
